# Sleep quality and its associated factors among women of reproductive age in Ethiopia: a systematic review and meta-analysis

**DOI:** 10.3389/fpsyt.2026.1550300

**Published:** 2026-05-20

**Authors:** Diriba Dereje Olana, Tesema Etefa Birhanu, Adisu Ewunetu Desisa

**Affiliations:** 1 Department of Biomedical Sciences, Faculty of Medical Sciences, Institute of Health, Jimma University, Jimma, Ethiopia; 2 Department of Public Health, Institute of Health Science, Wollega University, Nekemte, Ethiopia

**Keywords:** sleep disorder, sleep quality, determinants, factors, reproductive age women

## Abstract

**Background:**

Quality sleep is vital for women’s health during reproductive years, affecting both physical and mental well-being. In Ethiopia, socio-economic and cultural factors worsen sleep issues, but data on this demographic are scarce. This systematic review and meta-analysis assesses the prevalence of poor sleep quality among Ethiopian women and identifies contributing factors, aiming to inform interventions and policies to improve sleep health in low-resource settings.

**Method:**

This systematic review followed PRISMA guidelines and searched PubMed, Scopus, and Web of Science for observational studies. We included studies utilizing the Pittsburgh Sleep Quality Index (PSQI), as it is the most widely validated tool for assessing subjective sleep quality across diverse populations. Reviewers independently screened articles using Rayyan and assessed study quality with the Joanna Briggs Institute tools. Data were analyzed using Stata version 17. To account for potential clinical and methodological variability across studies, a random-effects model was employed to pool results, with heterogeneity assessed using statistics and the Cochrane’s Q test. Publication bias and sensitivity analyses were also performed.

**Result:**

Nine studies involving 4,376 women of reproductive age (15–49 years) in Ethiopia were included. The pooled prevalence of poor sleep quality was 49.17% (95% CI: 35.29, 63.08). Significant predictors of poor sleep quality included intimate partner violence (OR: 3.24), depression (OR: 3.37), unplanned pregnancy (OR: 2.71), multigravidity (OR: 2.61), and substance use (OR: 2.24).

**Conclusion:**

A systematic review indicates that nearly half of Ethiopian women of reproductive age experience poor sleep quality. Key factors include unplanned pregnancies, substance use history, intimate partner violence, previous depression, stress, being in the third trimester, and comorbidities; these need urgent attention and the implementation of screening and preventive measures. Future research should focus on effective interventions to improve sleep quality in these populations.

**Systematic Review Registration:**

https://www.crd.york.ac.uk/prospero/, identifier CRD42023455867.

## Introduction

Sleep quality is an essential factor in physiological and psychological recovery and is significantly influenced by the bed system and environmental factors. The Pittsburgh Sleep Quality Index (PSQI) is a widely used tool for evaluating sleep quality over the past month. A global PSQI score greater than 5 indicates poor sleep quality, with total scores ranging from 0 to 21; higher scores indicate worse sleep quality. Specifically, a score above 5 indicates compromised sleep quality. Sleep quality is crucial in overall health and wellness, especially for women of reproductive age (1). The World Health Organization highlights the crucial role of quality sleep in overall physical, mental, and emotional well-being (2, 3). Poor sleep quality can result in numerous health problems (4). Ethiopia faces unique socio-economic and cultural factors affecting sleep quality, including high poverty rates, limited access to healthcare, and varying cultural attitudes towards sleep and health (5, 6).

Despite growing recognition of sleep as an essential aspect of health, comprehensive data on sleep quality among Ethiopian women of reproductive age (15–49 years) remains insufficient. The intersection of these factors and mental health issues like anxiety and depression highlights the need to examine sleep health in this demographic (7). This gap is particularly problematic, given that sleep disturbances can worsen existing health problems, such as anxiety and depression, which are widespread in this population (8, 9).

Understanding the prevalence of poor sleep quality and its factors is vital for identifying issues that can be targeted through interventions to improve health outcomes for women and their families. Inadequate sleep can negatively affect pregnancy, infant health, and maternal well-being, making it crucial to address these matters in maternal health programs. Poor sleep quality is associated with adverse reproductive health outcomes, such as challenges in conception, pregnancy complications, and adverse effects on fetal development. These insights can help policymakers and healthcare providers develop integrated strategies to enhance sleep health within the broader context of women’s health in the country (10, 11).

Poor sleep quality in women of reproductive age affects physical and mental health, reproductive outcomes, and overall quality of life (8). Inadequate sleep is linked to a higher likelihood of developing chronic conditions like obesity, diabetes, heart disease, and high blood pressure. Likewise, insufficient sleep can weaken the immune system, increasing vulnerability (12, 13). Sleep disruptions can disrupt hormonal regulation, leading to irregular menstrual cycles. Inadequate sleep quality during pregnancy is associated with adverse effects such as gestational diabetes, premature labor, and low birth weight. Additionally, it can influence maternal mental health after childbirth (14, 15). Inadequate sleep leads to daytime fatigue, affecting productivity and social interactions. Ongoing sleep issues can decrease life satisfaction and create a cycle of stress that worsens sleep quality. As a result, poor sleep leads to higher healthcare utilization and costs due to associated health problems (13).

Several factors have been identified as determinants of sleep quality among reproductive-age women, including age, pregnancy condition, history of substance use, intimate partner violence, gravidity, depression, stress, gestational age, comorbid diseases, palpable thyroid gland, premenstrual syndrome, family size, maternal education, medical disorders, history of maternal illness in the family, maternal occupation, sleep hygiene, and parity (8).

Despite a growing number of individual studies across Ethiopia, there remains a lack of a comprehensive national synthesis to reconcile varying prevalence rates and identify consistent risk factors. This systematic review and meta-analysis fills this gap by providing the first pooled evidence specifically for Ethiopian women of reproductive age. By consolidating fragmented regional data, this study identifies socio-demographic, psychological, and environmental predictors, offering a robust evidence base for policymakers. These insights are critical for developing targeted reproductive health interventions and guiding public health strategies in similar low-resource settings.

## Method

### Data sources and search strategy

The review process adhered to the Preferred Reporting Items for Systematic Reviews and Meta-Analyses (PRISMA) checklist. We retrieved potential articles from electronic databases, including PubMed, Scopus, and Web of Science. The search for articles employed free-text search terms and Medical Subject Headings (MeSH). We utilized the following search terms: “sleep quality,” “post-partum women,” “Pregnant women,” “reproductive age women,” “women attending antenatal care follow-up,” “breastfeeding women,” “Predictors,” “Factors,” “Risk factors,” “Prevalence,” “Proportion,” and “Ethiopia.” Boolean operators such as “AND” and “OR” were used in conjunction with truncations, with no restrictions on publication dates. A comprehensive literature search was conducted, and June 20, 2024, was selected as the final cutoff to capture all relevant studies published up to the last search execution. This timeframe ensures the inclusion of the most recent data available before commencing data synthesis and analysis. The search strategy was first developed for PubMed using a combination of keywords and MeSH terms: ((sleep quality) AND (pregnant mothers OR women attending antenatal care follow-up* OR reproductive age women* OR breastfeeding women* OR post-partum women*) AND (predictors OR risk factors[MeSH Terms] OR associated factors OR barriers[MeSH Terms]) AND (prevalence OR proportion [MeSH Terms]) AND (Ethiopia))**. This core strategy was then adapted to the specific syntax and indexing requirements of Scopus and Web of Science. To ensure brevity, we omitted the full search strings for all databases and included only the specific PubMed string. The study protocol was prospectively registered on PROSPERO (CRD42023455867) to ensure transparency and minimize the risk of reporting bias.

### Inclusion and exclusion criteria

We focused on observational studies that primarily assess sleep quality and explicitly target factors related to sleep quality among women of reproductive age (15–49 years), regardless of study period, underlying disease, or specific type of reproductive-age population. Our analysis excluded these: clinical trials, editorial letters, reviews, and commentaries. In the end, two reviewers (DDO and AED) independently assessed the studies for eligibility, and any differences were settled through discussion and agreement with a third reviewer (TEB).

### Study selection

All retrieved articles were initially exported to Zotero, a reference management software, to eliminate duplicate studies. Subsequently, the articles were imported into Rayyan for screening. Two review authors (DDO and AED) independently screened the articles based on their titles and abstracts. Next, the full text of potentially eligible articles was retrieved and evaluated against predetermined eligibility criteria. Any disagreements between the authors during the study screening were resolved by a third review author (TEB).

### Exposure

We extracted all factors significantly associated with poor sleep quality in the included studies.

### Outcome

The outcome we are going to see is poor sleep quality in Reproductive-age women.

### Outcome measurement

The primary focus of this review was to assess the prevalence of sleep quality among women of reproductive age. We included sleep quality evaluated using the Pittsburgh Sleep Quality Index (PSQI), a self-report questionnaire consisting of 19 items that examine seven components of sleep: subjective sleep quality, sleep latency, sleep duration, habitual sleep efficiency, sleep disturbances, daytime dysfunction, and the use of sleep medications. Each component is scored on a scale of 0 to 3, yielding a total global PSQI score of 0 to 21. A global score of ≥5 was considered indicative of poor sleep quality among reproductive-age women. The secondary outcome of this review explored the factors associated with sleep quality in this population in Ethiopia, which were measured using odds ratios (OR) indicated in the included study. The odds ratio for each identified factor was calculated from the binary outcome data reported in each study.

### Quality assessment

The chosen publications were rigorously assessed using the Joanna Briggs Institute Critical Appraisal Checklist for Meta-Analysis (JBI-MAStARI). This 9-item checklist covers sampling (Q1–Q3), description of subjects and setting (Q4), coverage of analysis (Q5), validity and reliability of condition measurement (Q6–Q7), statistical analysis (Q8), and response rate (Q9). For each study, reviewers recorded Y (yes) or NA (not applicable); each “Y” scored 1 point, and totals were summed to 9. Studies that scored more than six out of 10 were deemed high quality; those scoring 5 or 6 out of 10 were classified as good quality, while scores below five were considered poor quality. Items were judged from the methods/results sections, with disagreements resolved through discussion (or a third reviewer). The quality assessment was conducted independently by two review authors (AED, DDO), with any disagreements resolved by the third author (TEB). Studies with higher totals were considered to have a lower risk of bias.

### Data extraction

A standardized data extraction tool adapted from the Joanna Briggs Institute (JBI) was employed to extract data from the articles included in the review. Two authors (DDO and AED) independently gathered data from each primary study included in this meta-analysis. From each study, information such as the first author’s name, the study’s region and setting, the year of publication, the study design, the participants involved, the sampling technique, the sample size, prevalence, and factors associated with sleep quality, as well as measures of association (odds ratio), were collected.

### Statistical analysis

The extracted data were imported into STATA version 17 for analysis. Tables, figures, and forest plots were utilized to describe and summarize the findings. We calculated the I² statistic for each pooled analysis to assess study heterogeneity, which quantifies the percentage of total variation among studies; thresholds of 25%, 50%, and 75% indicate low, moderate, and high heterogeneity, respectively. The choice between models was based on the level of heterogeneity: a random-effects model was used when substantial heterogeneity was identified, while a fixed-effects model was applied when none was identified. We will also conduct subgroup and sensitivity (leave-one-out) analyses to explore sources of heterogeneity when high heterogeneity is detected. Publication bias was evaluated through a visual inspection of funnel plots and statistically verified using Egger’s regression test. However, evaluating publication bias for each factor was not feasible due to the limited number of studies available. We conducted a sensitivity analysis to see the effects of omitting each survey on the overall impact.

## Results

### Prevalence of poor sleep quality among women of reproductive age in Ethiopia

**Figure 1:**
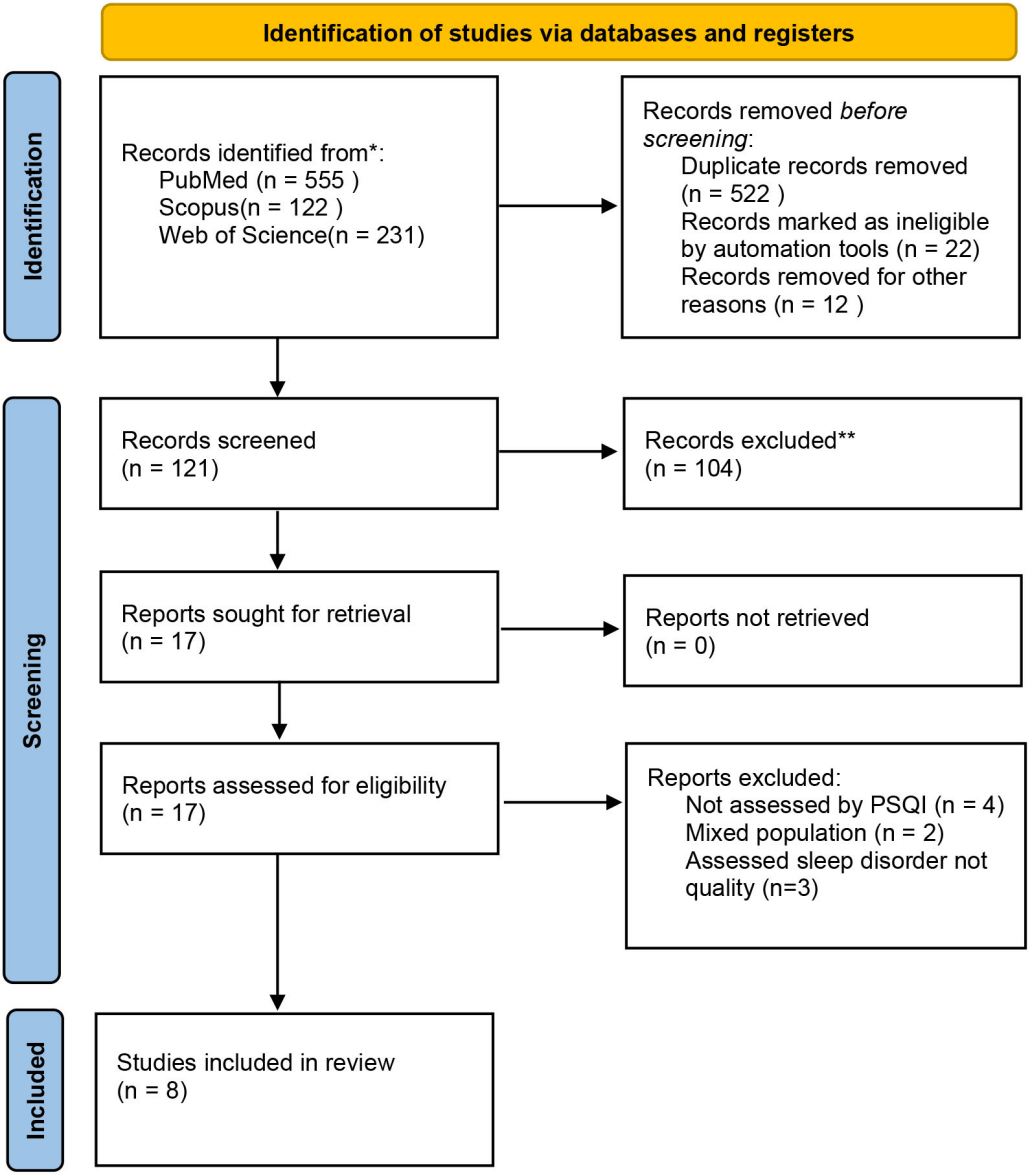
Prisma flow diagram of the study screening procedure.

**Figure 2:**
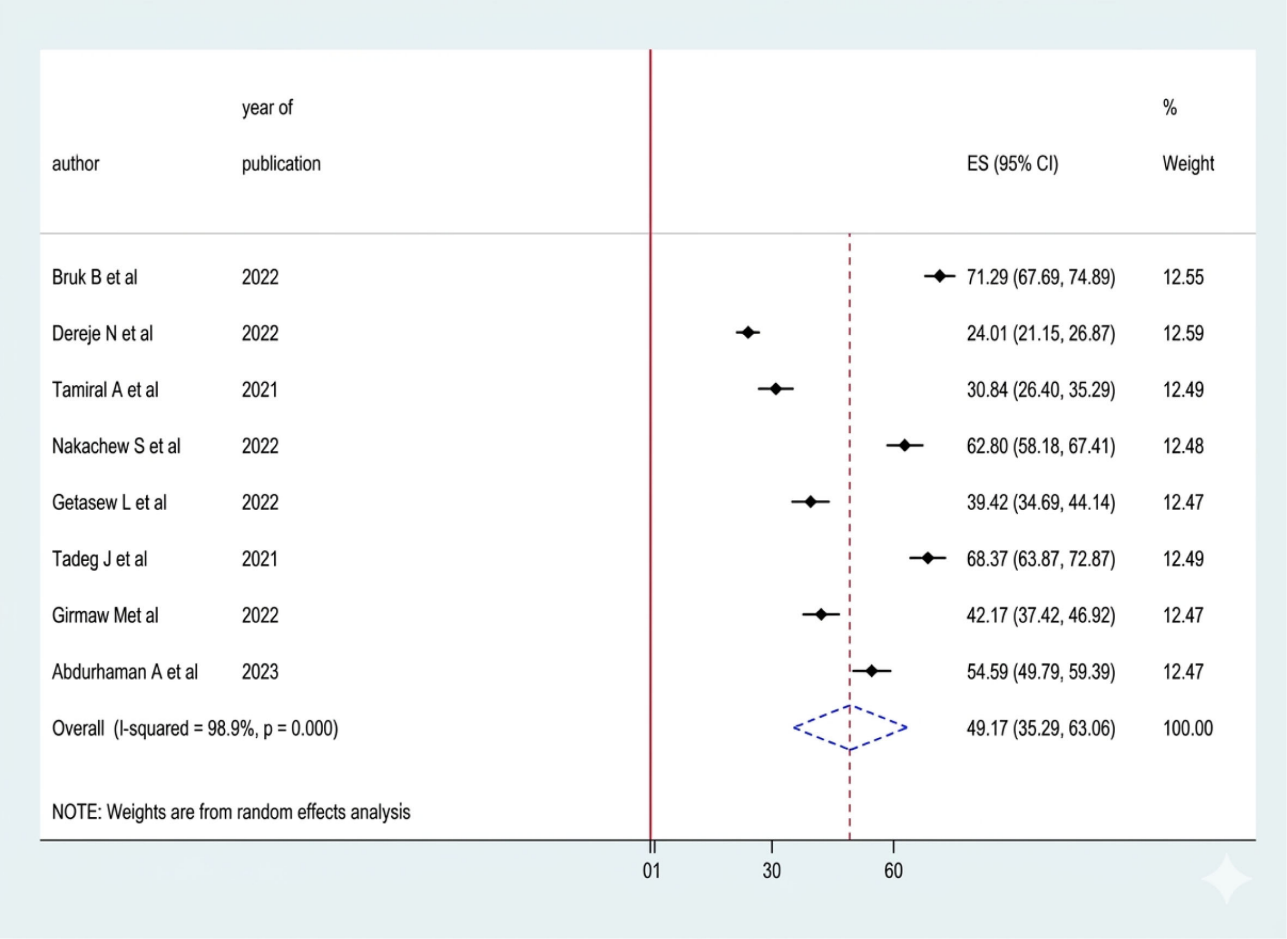
Pooled prevalence of sleep quality among RAW in Ethiopia.

**Figure 3:**
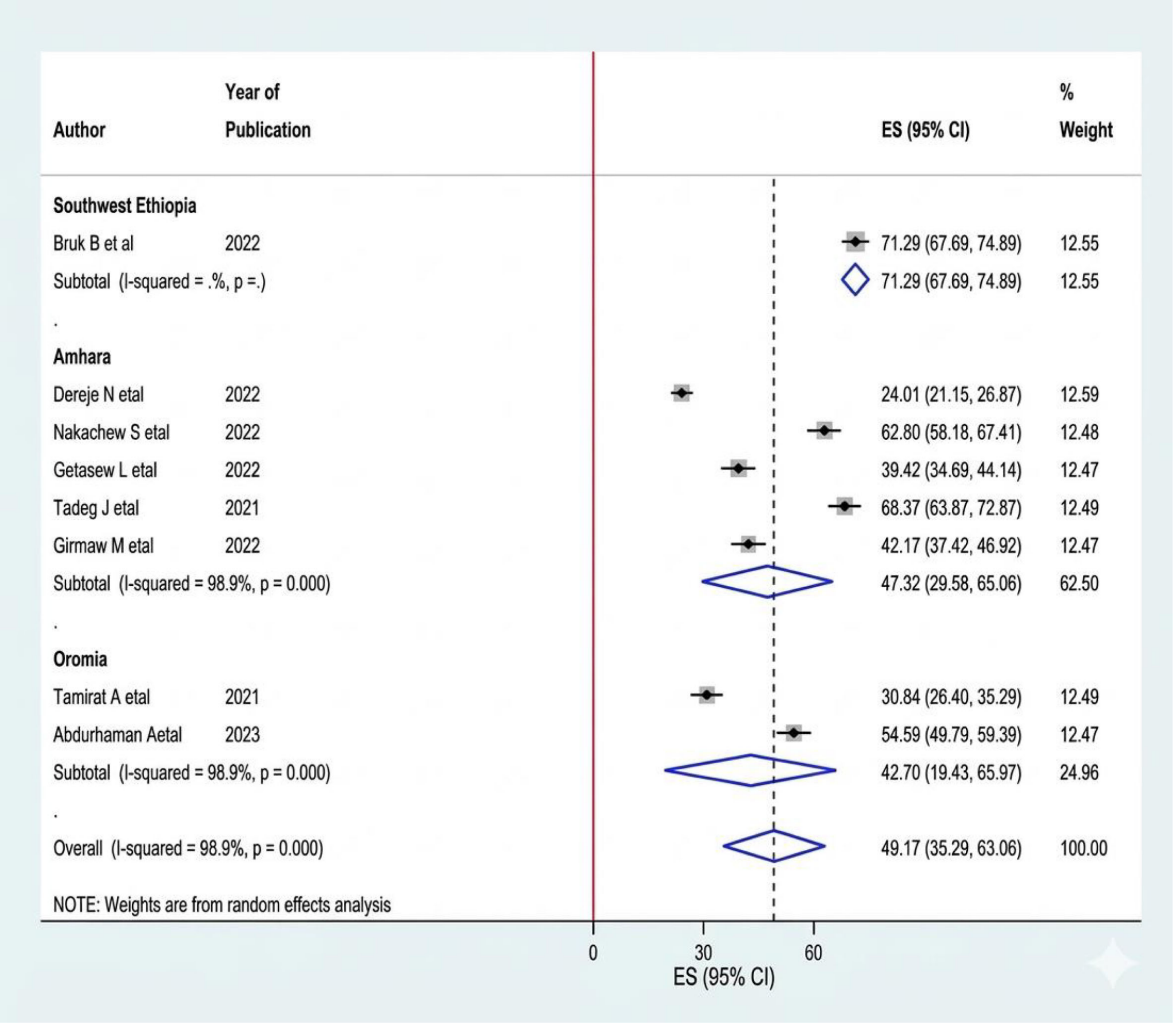
Subgroup analysis based on the region where the study was conducted on sleep quality among RAW in Ethiopia.

**Figure 4:**
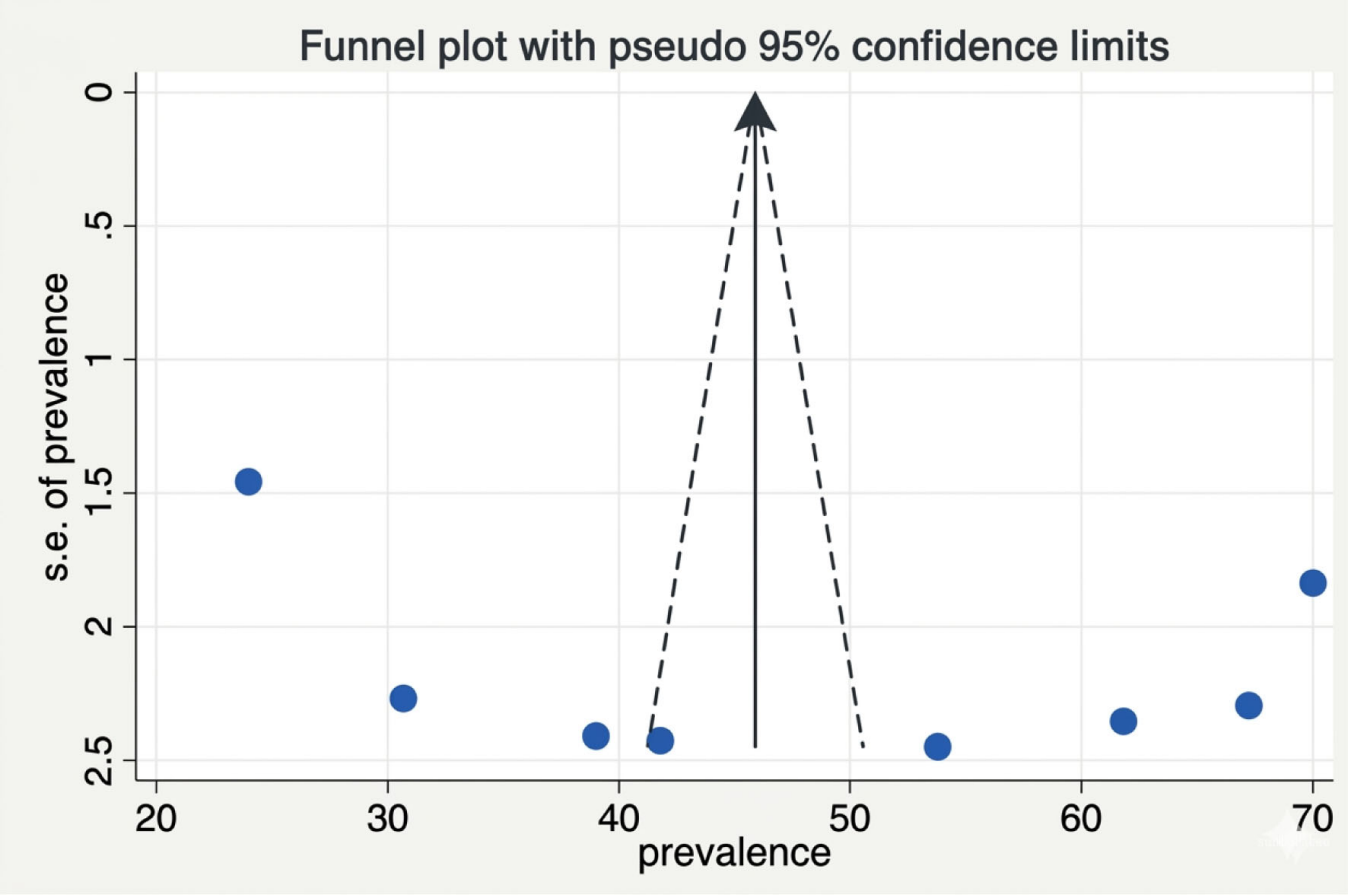
Funnel plot to check publication bias.

A systematic search of electronic databases and library catalogs identified 908 published articles. A total of 8 studies with a sample size of 4376 were included to determine the pooled prevalence of sleep quality among women of reproductive age in Ethiopia (**Figure 1**). Of these studies, two were conducted in the Oromia regional state (5, 16) Five of them were conducted in the Amhara regional state (10, 17–20). And one study was conducted in Southwest Ethiopia (8). Accordingly, the pooled prevalence of sleep quality among women of reproductive age was 49.17% (95% CI 35.29, 63.08) (**Figure 2**). The highest prevalence was observed in Southwest Ethiopia, 71.29 (95%CI: 67.69, 74.89), while the lowest was observed in the Oromia region, 42.70 (95%CI: 19.43, 65.97) (**Figure 3**). As indicated on the funnel plot in **Figure 4**, no significant publication bias was observed (**Table 1**).

**Table 2:** The joanna briggs institute critical appraisal tools (JBI) for included studies in use in JBI systematic reviews and meta-analysis.

Quality of the domain	Study author’s							
	Bruk B et al.	Dereje N et al.	Tamirat A et al	Nakachew S et al	Getasew L et al.	Tadeg J et al.	Girmaw M et al	Abdurhaman A et al.
Q1.Was the sample frame appropriate to address the target population?	Y	Y	Y	Y	Y	Y	Y	Y
Q2. Were study participants sampled appropriately?	Y	Y	Y	Y	Y	Y	Y	Y
Q3. Was the sample size adequate?	Y	Y	Y	Y	Y	Y	Y	Y
Q4. Were the study subjects and the setting described in detail?	Y	Y	Y	Y	Y	Y	Y	Y
Q5. Was the data analysis conducted with sufficient coverage of the identified sample?	Y	Y	Y	N	Y	Y	Y	Y
Q6. Were valid methods used for the identification of the condition?	Y	Y	Y	Y	Y	Y	Y	N
Q7. Was the condition measured in a standard, reliable way for all participants?	Y	Y	Y	Y	Y	Y	Y	Y
Q8. Was there an appropriate statistical analysis?	Y	Y	Y	Y	Y	Y	Y	Y
Q9. Was the response rate adequate, and if not, was the low response rate managed appropriately	Y	Y	NA	NA	Y	Y	NA	NA
Total score out of 9	9	9	8	8	9	9	8	7

### Sensitivity analysis and publication bias

**Figure 5:**
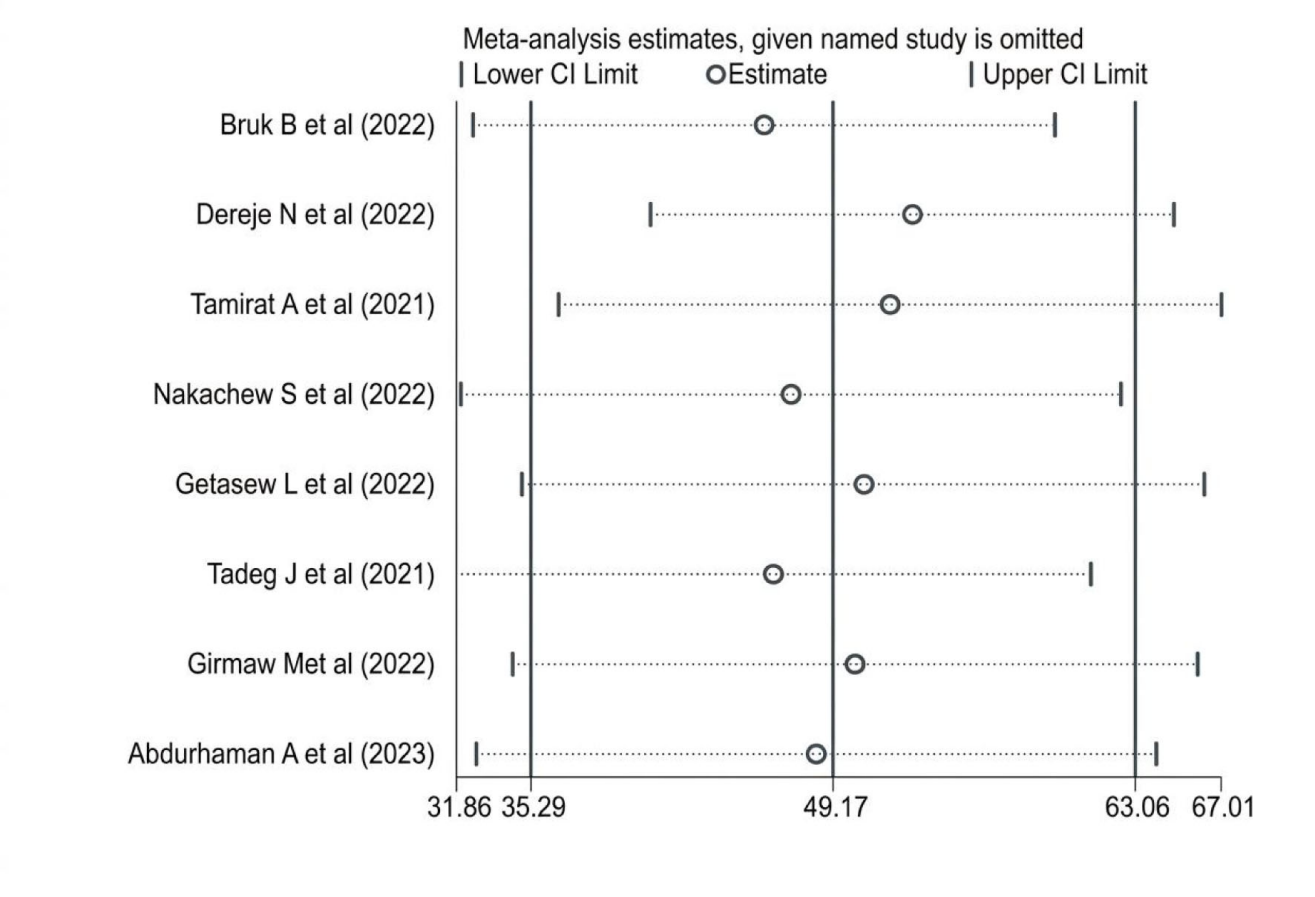
Sensitivity analysis on sleep quality among RAW in Ethiopia.

To evaluate the robustness of our findings and investigate potential sources of heterogeneity, we performed a sensitivity analysis using a random-effects model, systematically excluding each study in turn. This analysis aimed to identify any individual study or outlier that might disproportionately influence the pooled prevalence or odds ratios for sleep quality. The results demonstrated that no single study significantly altered the overall meta-analysis estimates, confirming the stability and reliability of our conclusions and no publication bias detected (**Figures 4**, **5**).

#### Subgroup analysis

Subgroup analysis by region was conducted to reduce potential random differences across studies. The highest prevalence was observed in Southwest Ethiopia, 71.29 (95% CI: 67.69, 74.89), while the lowest was observed in Oromia, 42.70 (95% CI: 19.43, 65.97) (**Figure 3**).

### Determinants of sleep quality

#### Association between the age of women and sleep quality

**Figure 6:**
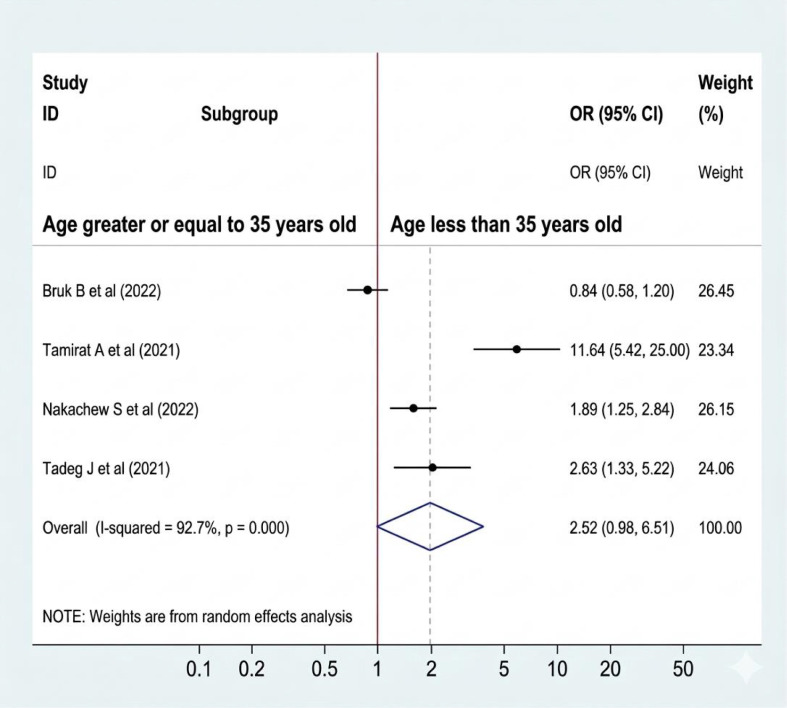
Association between the age of women and sleep quality in Ethiopia.

Out of 8 studies, four studies were included to look at the association between the ages of women and sleep quality for the final meta-analysis (8, 18, 19, 21). A random-effect model was used to estimate the pooled association between women’s age and sleep quality (I^2^ = 92.7%, P-value=0.000). Among the included studies, three have a significant association with sleep quality (18, 19, 21). While one of the included did not have a significant association (8). The pooled result of the analysis indicates that the age of women has no association with sleep quality (OR: 2.52, CI: 0.98, 6.51) (**Figure 6**). Although some pooled estimates were based on only two studies in the available literature, we included these results to synthesize current evidence and provide a foundation for future research. However, we acknowledge that this limits the robustness of those specific findings.

#### Association between the condition of pregnancy and sleep quality

**Figure 7:**
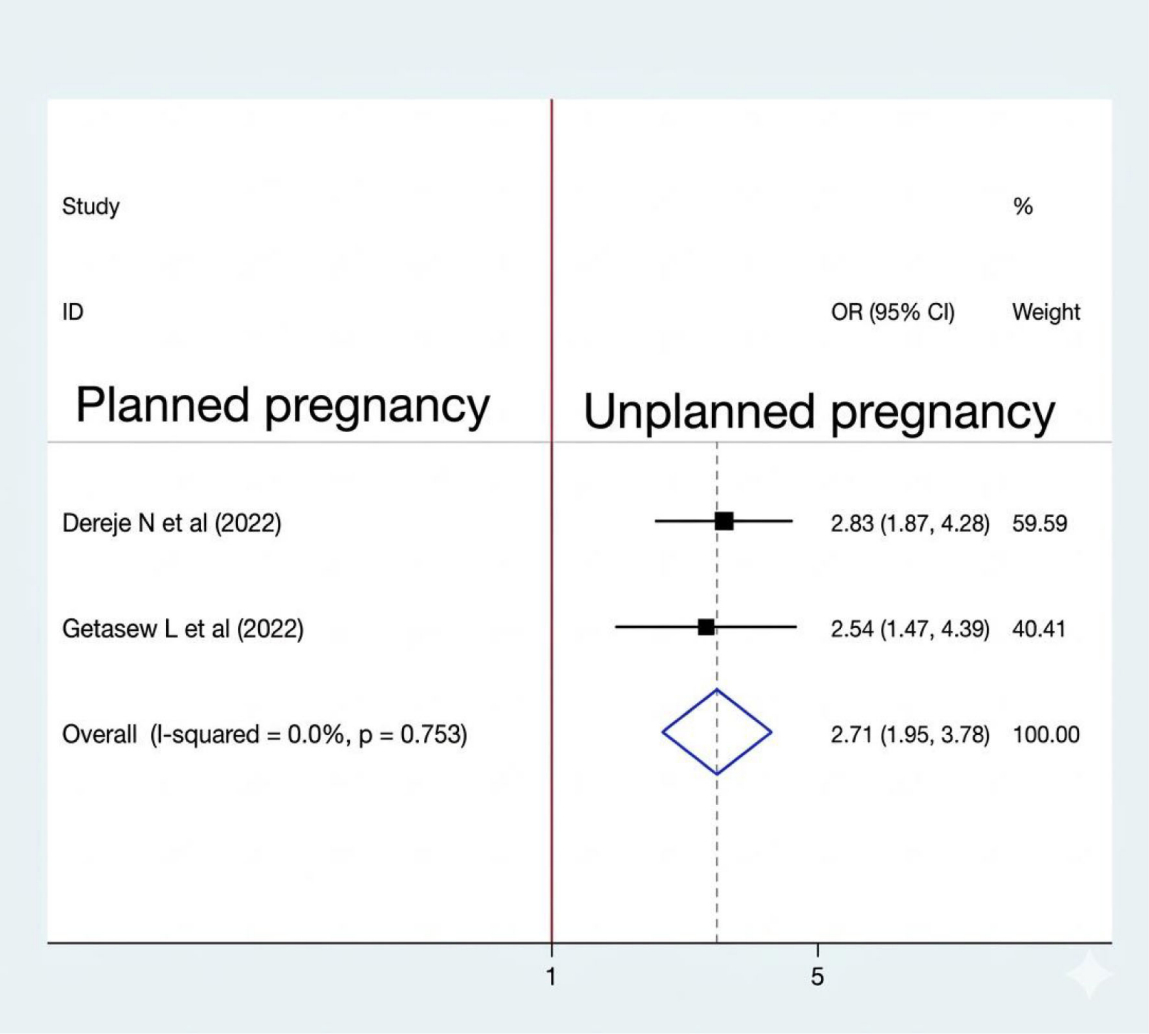
Association between pregnancy condition and sleep quality among RAW in Ethiopia.

The pooled analysis indicates that the odds of poor sleep quality were 2.71 times higher among women with an unplanned pregnancy than among their counterparts (OR: 2.71, CI: 1.95, 3.78). Among the eight studies, two were included to determine the association between pregnancy condition (planned or unplanned) and sleep quality among women of reproductive age (17, 20), and both were significantly associated with sleep quality. A fixed-effect model was used to estimate the pooled association between pregnancy condition and sleep quality (I2 = 0.00%, P-value = 0.753) (**Figure 7**).

#### Association between history of substance use and sleep quality

**Figure 8:**
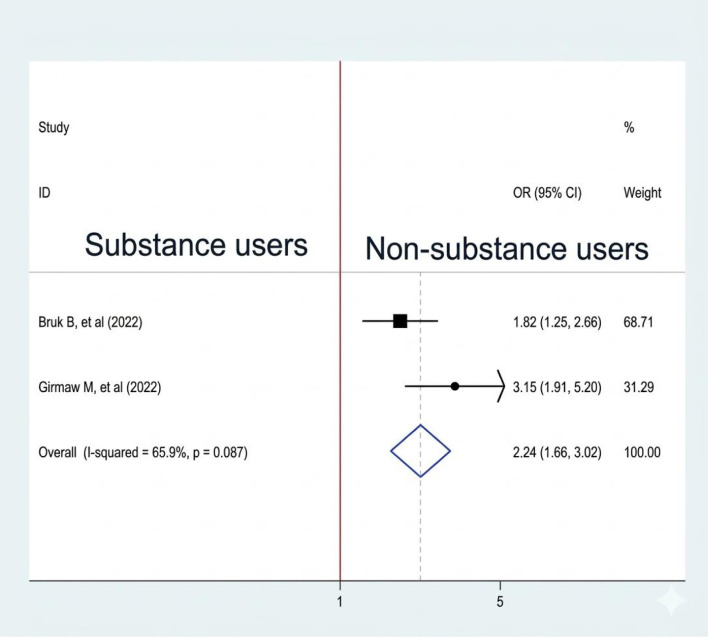
Association between substance use and sleep quality among RAW in Ethiopia.

Out of 8 studies, only two studies (8, 10). We included looking at the association between substance use and sleep quality among reproductive-age women, and both are significantly associated with sleep quality. A fixed-effect model was used to estimate the pooled association between substance use and sleep quality (I2: 65.9, p-value = 0.087). The pooled prevalence of the analysis result indicates that women who are substance users are 2.24 times more likely to experience poor sleep quality than non-substance users (OR: 2.24, CI: 1.66,3.02) (**Figure 8**).

#### Association between intimate partner violence and sleep quality

**Figure 9:**
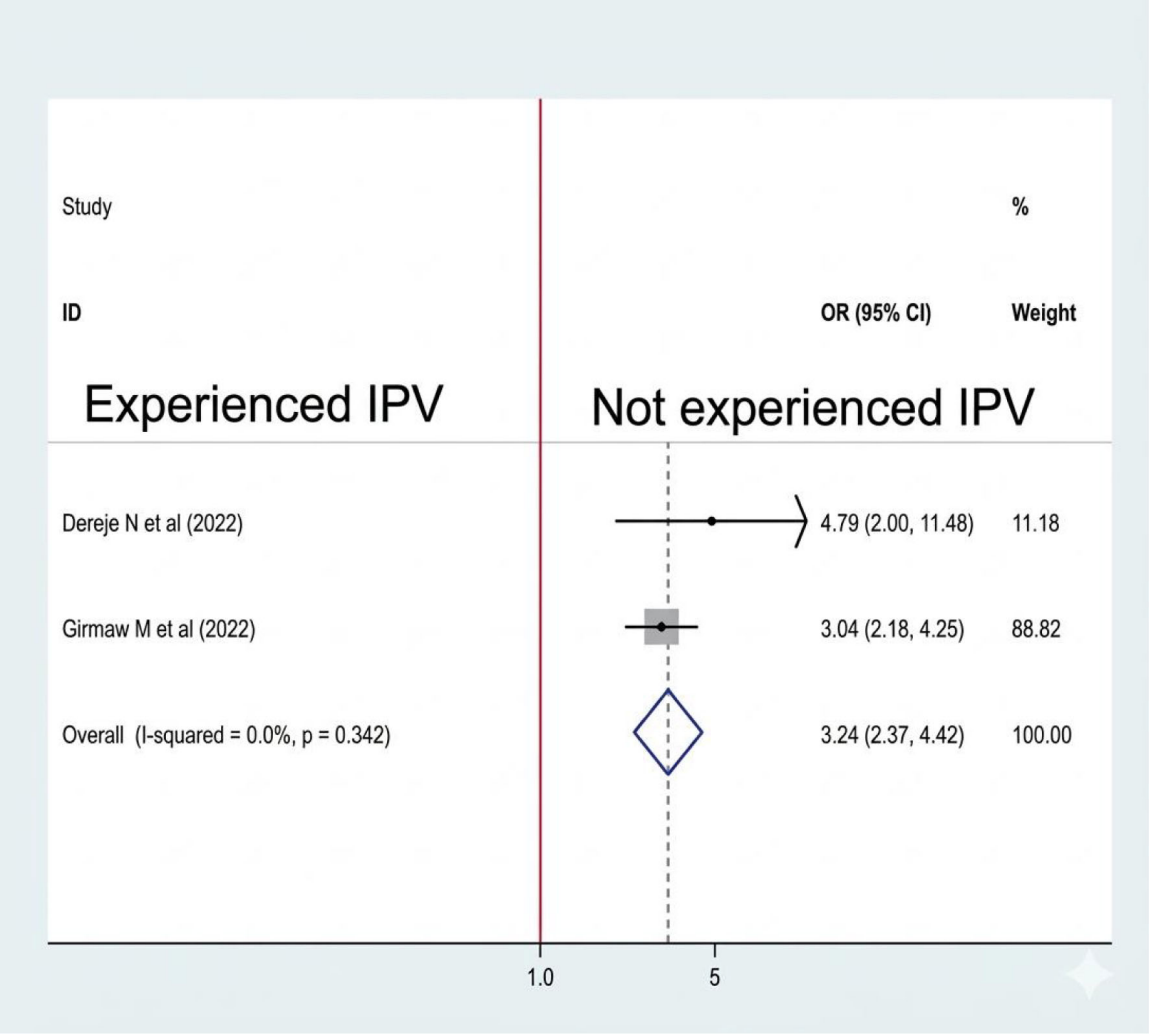
Association between IPV and sleep quality among RAW in Ethiopia.

Two studies (10, 17)We included them to see the association between intimate partner violence and sleep quality, and both of them were associated with sleep quality. A fixed-effect model was used to estimate the pooled association between intimate partner violence and sleep quality (I2: 0.00, p-value = 0.342). The pooled prevalence of the analysis result reveals that the odds of poor sleep quality were 3.24 times higher among women who had experienced intimate partner violence than their counterparts (OR: 3.24, CI: 2.37, 4.42) (**Figure 9**).

#### Association between gravidity and sleep quality

**Figure 10:**
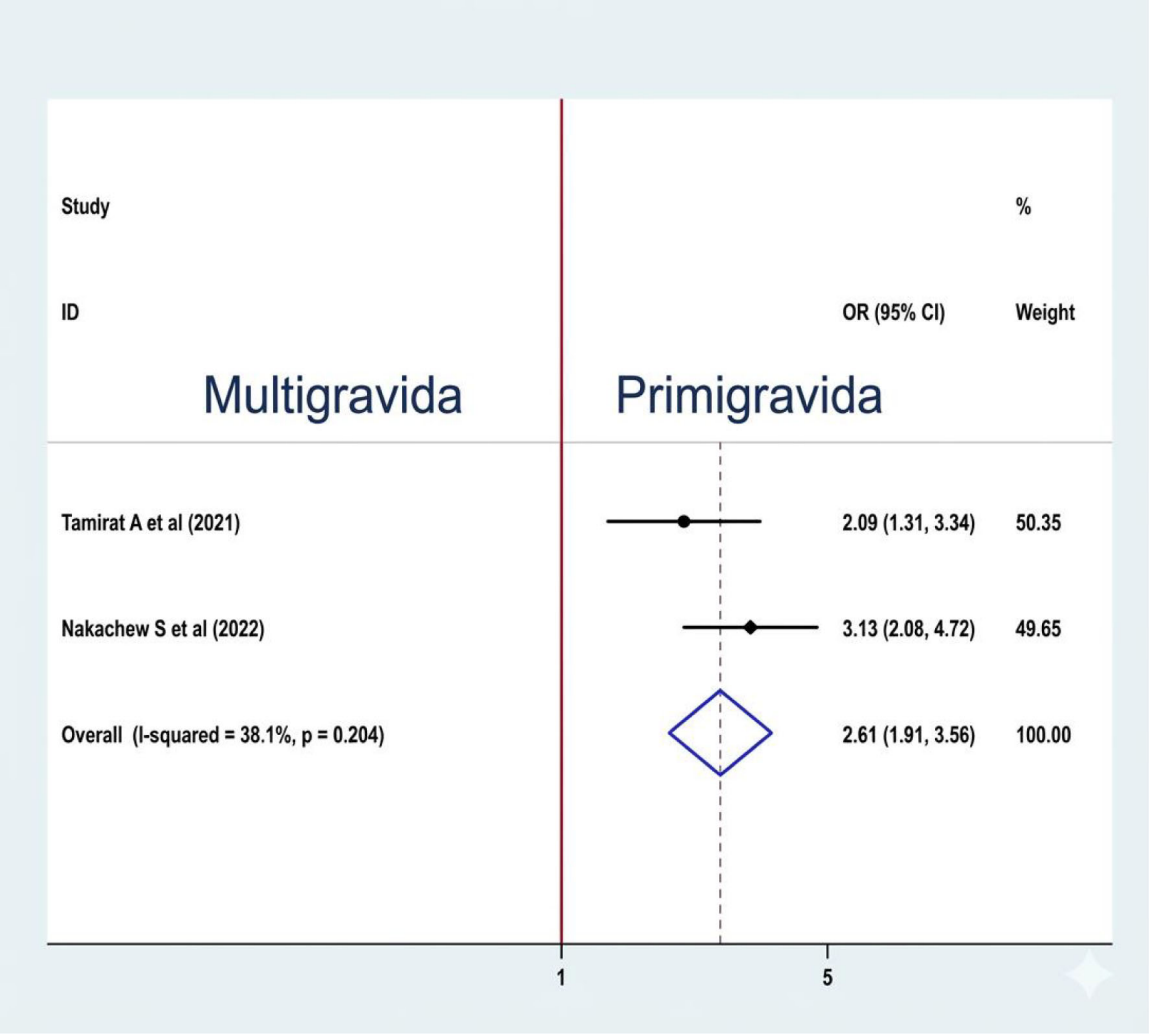
Association between gravidity and sleep quality among RAW in Ethiopia.

A fixed-effect model was used to estimate the pooled association between gravidity and sleep quality among reproductive-age women (I2: 38.1%, p-value: 0.204). Two studies (19, 21) were included to examine the association between the variables, and both were significantly associated with sleep quality. The pooled prevalence of the analysis result indicates that the odds of poor sleep quality are 2.61 times higher among multigravida women than their counterparts (OR: 2.61, CI: 1.91, 3.56) (**Figure 10**).

#### Association between depression and sleep quality

**Figure 11:**
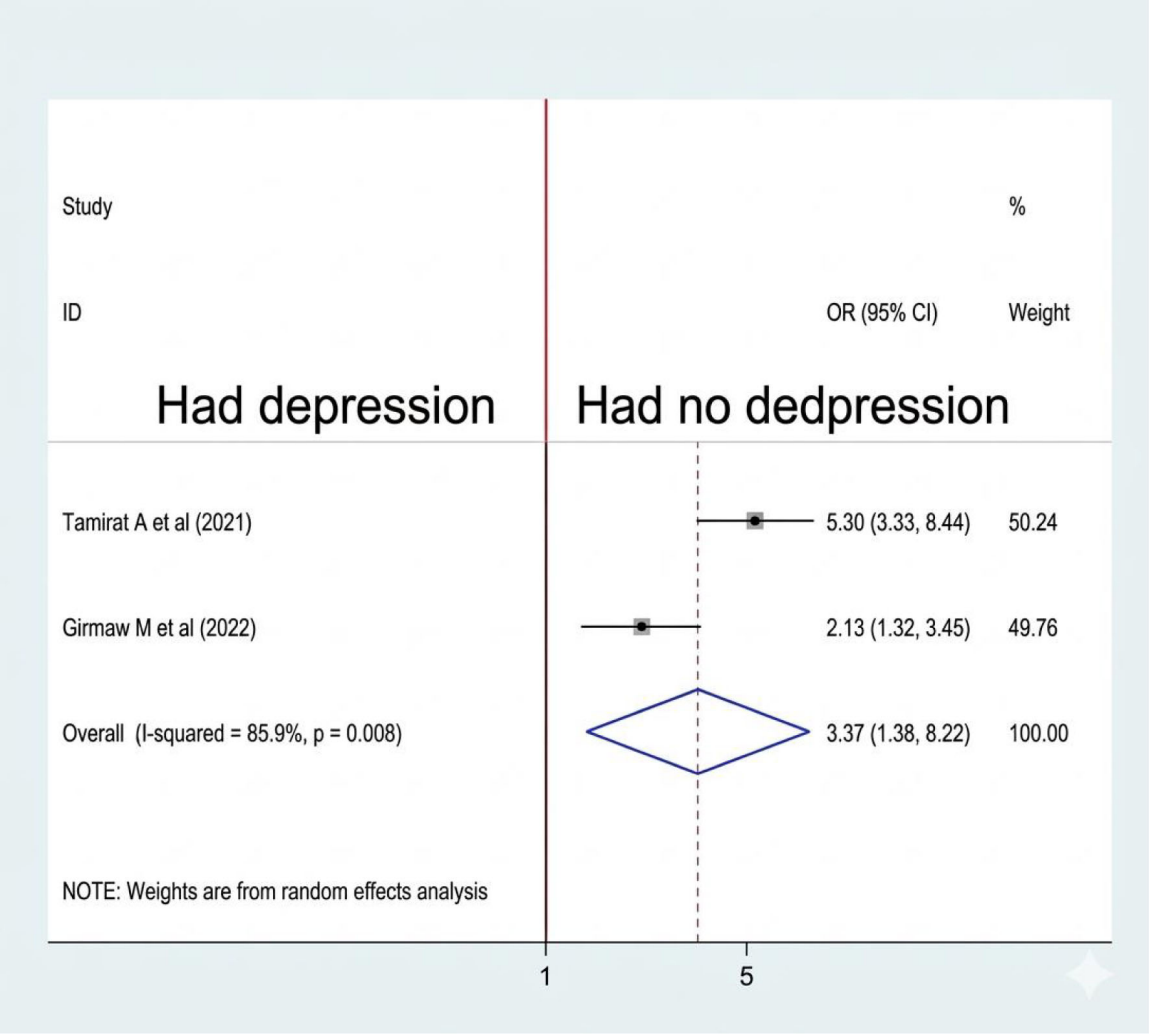
Association between depression and sleep quality among RAW in Ethiopia.

From 8 studies, we included two (10, 21) that examined the association between depression and sleep quality. Both the included studies were significantly associated with sleep quality. A random-effects model was used to estimate the pooled association between depression and sleep quality (I2: 85.9%; p-value: 0.008). The pooled prevalence of the analysis result indicates that women who had experienced depression were 3.37 times more likely to have poor sleep quality than their counterparts (OR: 3.37, CI: 1.38,8.22) (**Figure 11**).

#### Association between stress and sleep quality

**Figure 12:**
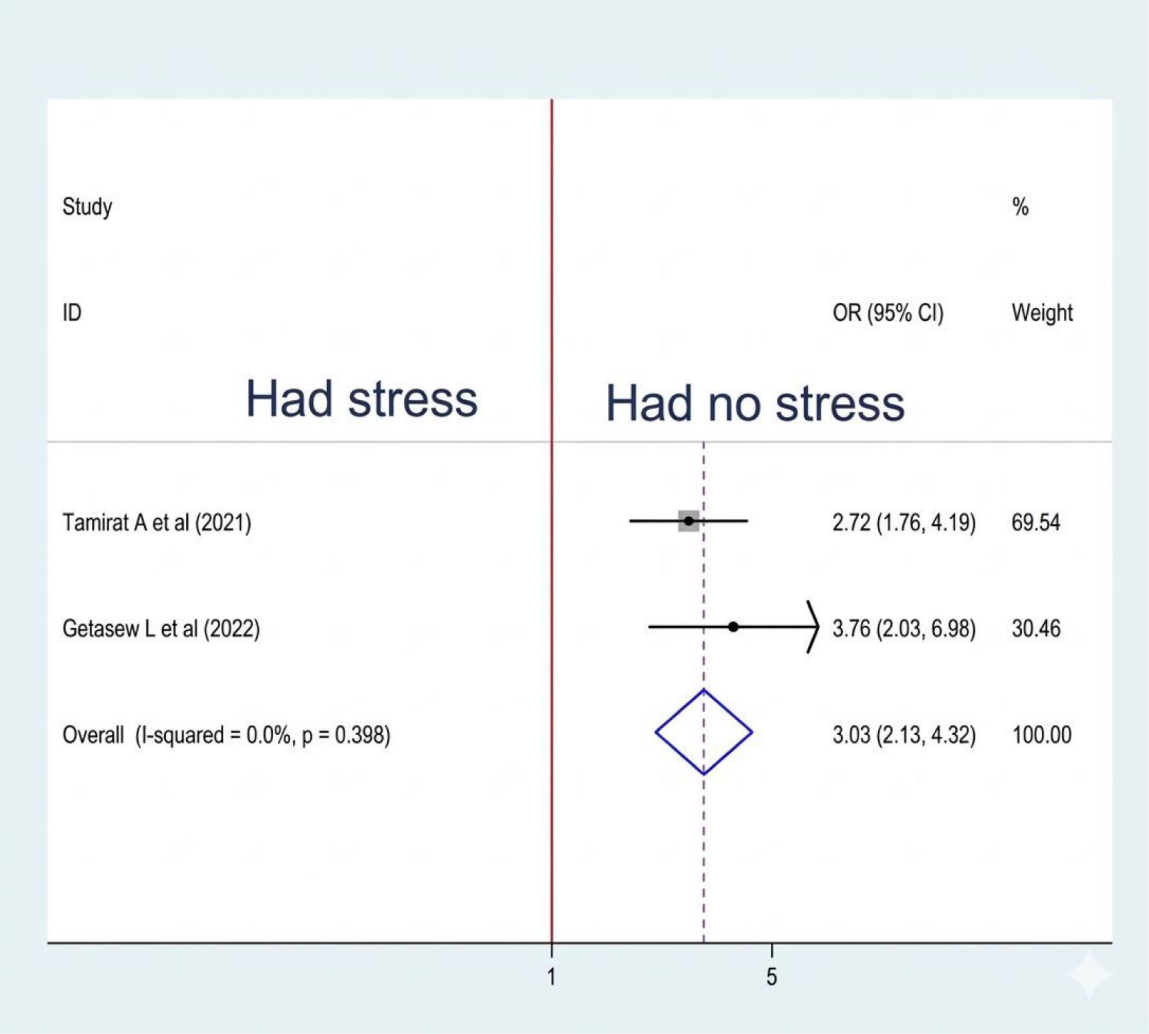
Association between stress and sleep quality among RAW in Ethiopia.

Two studies were included to examine the association between stress and sleep quality, and both showed significant associations (20, 21). A fixed effect model was used to estimate the pooled association between stress and sleep quality (I2: 0.0%, p-value=0.398). The pooled prevalence of the analysis indicates that the odds of poor sleep quality were 3.03 times higher among women who had experienced stress than their counterparts (OR: 3.03, CI: 2.13, 4.32) (**Figure 12**).

#### Association between gestational age and sleep quality

**Figure 13:**
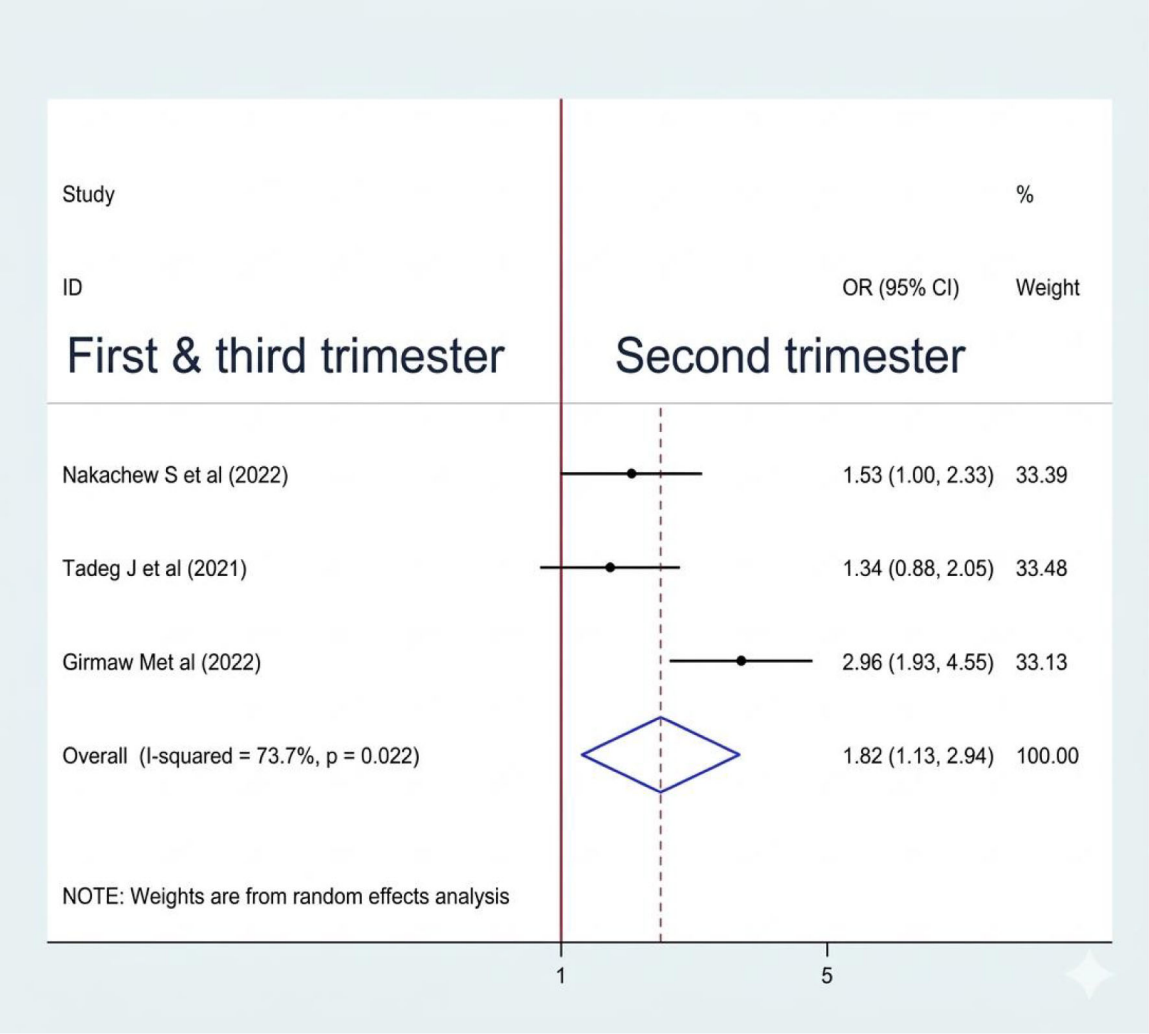
Association between gestational age and sleep quality among RAW in Ethiopia.

Of 8 studies, 3 (10, 18, 19) were included to examine the association between gestational age and sleep quality in women. Among the included studies, two (10, 19) were significantly associated with sleep quality, whereas one (18) was not. A random effect model was used to estimate the pooled association between gestational age and sleep quality (I2: 73.7%, p-value=0.022). The pooled prevalence of the analysis result indicates that the odds of poor sleep quality were 1.82 times higher among women who were in the first and third trimester when compared to those in the second trimester (1.82 (1.13, 2.94) (**Figure 13**).

#### Association between comorbid disease and sleep quality

**Figure 14:**
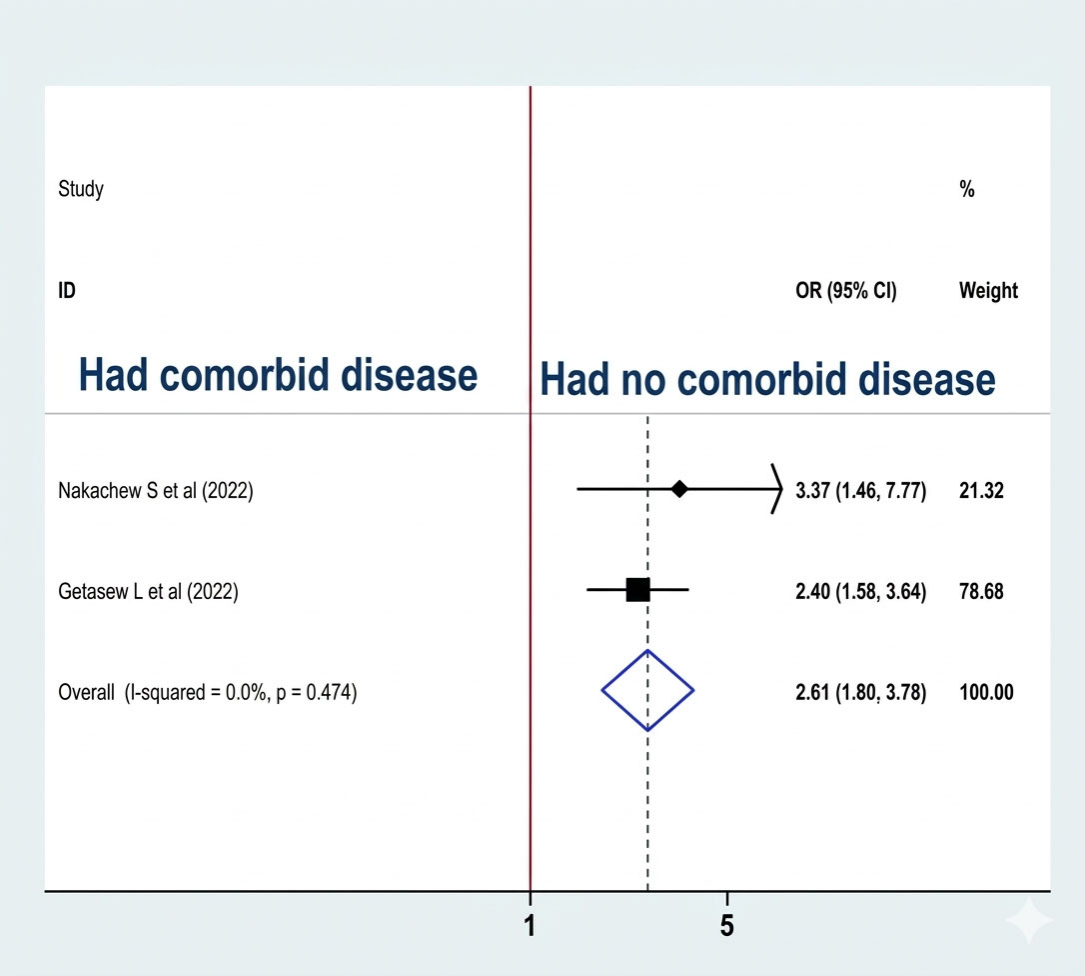
Association between comorbid disease and sleep quality among RAW in Ethiopia.

Two studies (8, 20) were included to examine the association between comorbid disease and sleep quality, and both studies showed significant associations. A fixed-effect model was used to estimate the pooled association between the availability of comorbid diseases and sleep quality (I2: 0.0%, p-value = 0.474). The pooled prevalence analysis indicates that the odds of developing poor sleep quality were 2.61 times higher among women with comorbid disease than among their counterparts (OR: 2.61, CI: 1.80, 3.78) (**Figure 14**).

### Variables that have an association in a single literature source

**Figure 15:**
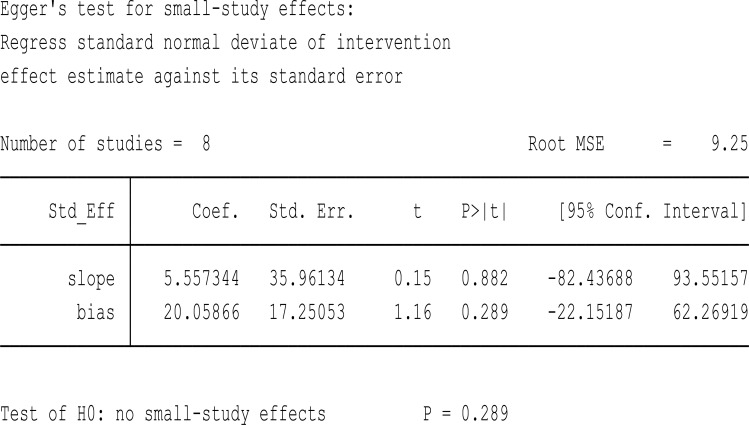
Egger’s test statistics to check the significance of publication bias.

Among the studies included in this study, some variables were significantly associated with sleep quality in a single study but were not included in the systematic review and meta-analysis. These include palpable thyroid gland and premenstrual syndrome reported in (8), family size, maternal education, medical disorder, history of maternal illness in the family, and maternal occupation are reported in (17) whereas sleep hygiene (20) and parity (18) were also reported as having a significant association in a single literature mention. On top of funnel plot, there is statistical significance of publication bias (**Figure 15**).

### Quality assessment

Most studies scored 7–9/9, showing generally higher quality. Minor limitations included one study with incomplete analysis coverage, one with unclear condition identification, and several with response-rate issues. Overall, the evidence is of high quality, with a few study-specific issues to consider (see **Table 2**).

**Table 1:** Abstraction of studies included in determining the prevalence of sleep quality among women of reproductive age group in Ethiopia, 2024.

Author	Year of publication	Region	Study design	Study area	Sample size	Prevalence
Bogale et al. (8)	2022	Southwest Ethiopia	Cross sectional	Mizan Aman Town	606	68.14(64.51, 71.77)
Gessesse, D.N.et.al (17).	202	Amhara	Cross sectional	Gondar City	858	24.01(21.15, 26.87)
Anbesaw T et al. (21)	2021	Oromia	Cross sectional	Jimma Town (at Jimma Medical Center)	415	30.26(25.88, 34.64)
Amare et al (19)	2022	Amhara	Cross sectional	Debra Berhan Town	422	62.65(58.04, 67.26)
Legas G et al. (20)	2022	Amhara	Cross sectional	Four selected referral Hospitals of the Amhara Region	411	38.30(33.67, 42.93)
Jemere et al (18)	2021	Amhara	Cross sectional	Wadila Primary Hospital in Kone Town	411	66.59(62.09, 71.089)
Takelle et al (10)	2022	Amhara	Cross sectional	University of Gondar Comprehensive Specialized Hospital	415	41.37(36.68, 46.07)
Abdurhaman A et al. (16)	2023	Oromia	Cross sectional	Referral hospitals in Oromia Regional State	414	53.43(48.67,58.18)

## Discussion

This meta-analysis aimed to estimate the prevalence of poor sleep quality and its associated factors among reproductive-age women. Notably, it represents the first comprehensive assessment of the pooled prevalence of poor sleep quality and related factors among reproductive-age women in Ethiopia. The review revealed significant variation in poor sleep quality among these women, with prevalence rates ranging from 24.01% to 68.14%, and a pooled prevalence of 49.17% (95% CI 35.29, 63.08). The findings regarding poor sleep quality among reproductive-age women in this study are consistent with those observed in other reviews conducted in China, which reported a pooled prevalence of 54.2% (11). While no other meta-analysis addresses this issue, the inadequate sleep quality reported by reproductive-aged women in this study aligns with findings from other Canadian research (22), Brazil (23), and the USA (24).

A current meta-analysis found that women of reproductive age with unplanned pregnancies had 2.7 times higher odds of experiencing poor sleep quality compared to those with planned pregnancies. This finding is supported by a study conducted in Cameroon (25) and China (26). Heightened psychological stress, anxiety, and uncertainty about the future often accompany unplanned pregnancies. Women may worry about financial stability, relationship dynamics, and their ability to care for a child. These concerns can disrupt sleep patterns, leading to insomnia and other sleep disturbances, and may negatively impact mental health, intensifying sleep-related issues (27–30).

Women who are substance users are 2.24 times more likely to experience poor sleep quality than non-substance users; this is supported by other studies conducted in China (31). Substance use significantly affects sleep quality. Alcohol, nicotine, and illicit drugs disrupt the natural sleep cycle, leading to poor rest. While alcohol may initially induce drowsiness, it often results in fragmented sleep. Stimulants like nicotine and cocaine hinder the ability to fall asleep and reduce sleep duration. Additionally, substance use can increase anxiety and stress, worsening sleep disorders and impacting overall health (32–34).

Our analysis results reveal that the odds of poor sleep quality were 3.24 times higher among women who had experienced intimate partner violence than their counterparts. Similarly, in a study done in Peru (35) and Portugal (36), intimate partner violence was associated with poor sleep quality in women. Intimate partner violence negatively affects sleep quality due to psychological and physiological factors. Survivors often face increased stress, anxiety, and post-traumatic stress disorder (PTSD), leading to disrupted sleep and insomnia. Fear and hypervigilance related to abuse make restful sleep difficult, while intrusive thoughts worsen the situation. Research shows that women experiencing IPV report significantly higher sleep disturbances, emphasizing the link between IPV and poor sleep quality (37, 38).

The odds of poor sleep quality are 2.61 times higher among multigravida women than their counterparts. Similarly, the same is true in a study done in China (39), Egypt (40), and India (41). Poor sleep quality in multigravida women can arise from factors like physical discomfort due to weight gain and fetal movement, hormonal changes, and anxiety about parenting. Additionally, frequent urination, sleep disorders like sleep apnea, and fatigue from caring for multiple children further disrupt sleep (42).

The pooled prevalence of the analysis result indicates that women who had experienced depression were 3.37 times more likely to have poor sleep quality than their counterparts. Similarly, this is true of studies conducted in China (43), Nepal (44), Saudi Arabia (45), and Australia (46). It disrupts sleep architecture, leading to decreased REM sleep and increased frequency of awakenings. The fatigue from depression can result in insomnia or excessive sleep, while negative thought patterns can exacerbate these issues, creating a cycle that worsens both mood and sleep (47, 48).

The pooled prevalence of the analysis indicates that the odds of poor sleep quality were 3.03 times higher among women who had experienced stress than their counterparts. In previous studies conducted in Jordan (49) and France (50), stress associated with poor sleep quality. Stress harms sleep quality by triggering the body’s stress response and releasing hormones like cortisol, which disrupt sleep patterns. Elevated stress often increases anxiety and racing thoughts, making relaxation difficult. Physical symptoms, such as muscle tension, can also hinder sleep. This cycle of anxiety and poor sleep can lead to a decline in overall well-being (51, 52).

The pooled analysis indicates that the odds of poor sleep quality were 1.82 times higher in the first and third trimesters than in the second trimester. In line with our study, the third trimester is associated with poor sleep quality in studies conducted in Japan (53) and China (54). In the third trimester, poor sleep quality is mainly due to physical discomfort from an enlarging belly, frequent urination, and fetal movements. Hormonal changes also contribute to insomnia and restless legs syndrome. Anxiety about labor and parenting, along with increased discomfort, further disrupts sleep (26, 40).

The pooled prevalence of the analysis result indicates that the odds of developing poor sleep quality were 2.61 times higher among women who had comorbid disease than their counterparts. In another study, comorbid disease is associated with sleep disorders (55). Comorbid diseases can significantly impact sleep quality in women by causing discomfort, pain, and fatigue. Chronic conditions like diabetes and cardiovascular disease can lead to issues such as sleep apnea. Additionally, mental health disorders like anxiety and depression can worsen sleep disturbances, with the stress of managing multiple health issues further affecting sleep. These reflect Ethiopia’s unique sociocultural landscape, where poor sleep is intensified by social stigma surrounding unplanned pregnancy and intimate partner violence, as well as the stimulant effects of culturally ingrained Khat and coffee consumption. Additionally, the high domestic workload and limited access to mental health and chronic care within the healthcare system leave Ethiopian women with fewer resources to manage the stressors that disrupt sleep (56, 57). In addition, poor sleep quality directly increases the headache-related impact in patients with migraine and tension-type headaches (TTH), as well as indirectly by increasing headache frequency and severity in migraine patients (49).

### Limitation

This study has several limitations that should be acknowledged. Firstly, the reliance on cross-sectional observational studies may introduce bias, as causality cannot be firmly established. Additionally, the variation in study designs and measurement tools across the included studies may affect the consistency and comparability of the findings. The lack of longitudinal data restricts insights into how sleep quality changes over time and its long-term implications for health outcomes. We noted that previous studies did not address headache, and we recommend that future researchers do so. Furthermore, some studies had relatively small sample sizes, which may limit the generalizability of their results to the wider population. The studies also varied in geographical focus, which could lead to regional disparities in sleep quality being overlooked. On top of that, we acknowledge that our assessment of sleep quality was based on the Pittsburgh Sleep Quality Index (PSQI), a self-reported, subjective measure with several limitations, including potential reporting bias. Therefore, we strongly recommend that future researchers consider employing objective assessment methods, such as polysomnography, to obtain a more accurate evaluation of sleep quality. We also acknowledge the possibility of residual confounding, as not all potential influencing factors could be controlled for in the original studies. Although the sensitivity analysis confirms the stability of our primary findings, the reported high heterogeneity and the limited number of studies for certain determinants warrant cautious interpretation. These specific results serve as a foundational baseline for future research rather than definitive conclusions, highlighting the need for more primary studies to improve the robustness of these estimates.

Lastly, potential publication bias could influence the findings, as studies with significant results are more likely to be published, potentially skewing the overall prevalence estimates. These limitations suggest that further research is needed to validate these findings and explore the underlying causes of poor sleep quality in this demographic.

## Conclusion

Nearly half of reproductive-age women experience poor sleep, linked to factors like unplanned pregnancy, IPV, and mental health stressors. To address this, maternal health clinics should integrate routine sleep screening into existing services, while public health policies in low-resource settings should prioritize low-cost sleep hygiene education. However, our findings are based on cross-sectional, self-reported data (PSQI) and on a limited number of studies for certain factors, which require cautious interpretation. Future research should focus on objective measures and culturally tailored interventions to strengthen these foundational conclusions.

## Data Availability

The original contributions presented in the study are included in the article/supplementary material. Further inquiries can be directed to the corresponding author.
